# Longitudinal associations between community violence exposure, posttraumatic stress symptoms, and eating disorder symptoms

**DOI:** 10.1186/s40337-024-00965-6

**Published:** 2024-01-11

**Authors:** Martina Isaksson, Johan Isaksson, Mary Schwab-Stone, Vladislav Ruchkin

**Affiliations:** 1 Child and Adolescent Psychiatry Unit, Department of Medical Sciences, Uppsala University, S-751 85 Uppsala, Sweden; 2 Center of Neurodevelopmental Disorders (KIND), Centre for Psychiatry Research, Department of Women’s and Children’s Health, Karolinska Institutet and Stockholm Health Care Services, Region Stockholm, Sweden; 3 Child Study Center, Yale University School of Medicine, New Haven, USA; 4 Sala Forensic Psychiatric Clinic, Sala, Sweden

**Keywords:** Eating disorders, Posttraumatic stress disorders, Community violence, Gender, Ethnicity, Mediation analysis, Moderation analysis, Longitudinal studies

## Abstract

**Background:**

Eating disorder (ED) symptoms have been associated with different types of traumatic events, such as exposure to sexual and physical violence, and emotional abuse. However, the relation between ED symptoms and community violence exposure (CVE) is underexplored, despite the latter’s adverse effects on many aspects of adolescent functioning. The primary aim of this study was to evaluate the relation between CVE and ED symptoms in adolescents, while also investigating the potential mediating and moderating roles of posttraumatic stress (PTS) symptoms, gender, and ethnicity.

**Methods:**

Data were collected longitudinally over two consecutive years in the city of New Haven, CT, in the United States. Participants were 2612 adolescent students from the public school system (1397 girls and 1215 boys) with an average age of 12.8 years (*SD* = 1.29). The students were comprised of several different ethnic groups, including Caucasians, African Americans and Hispanic Americans. Associations between CVE (no exposure, witnessing, and victimization) and PTS symptoms at year one, and ED symptoms (thoughts and compensatory behaviors) at year two, were assessed with self-rating instruments. Moderation and mediation analyses were conducted using a variant of linear regression (Hayes PROCESS macro).

**Results:**

ED symptoms at year two were significantly associated with both witnessing and being a victim of community violence at year one, with most or all of the relations being explained by PTS symptoms. Overall, neither gender nor ethnicity had a meaningful moderating effect in the observed relations.

**Conclusions:**

The findings support the notion that assessing and addressing PTS symptoms might be beneficial when treating individuals with ED symptoms who have experienced community violence, irrespective of gender or ethnicity.

## Abstract (plain language summary)

In this study we wanted to see if there was a relation between adolescents experiencing violence in their community and showing signs of eating disorders. We also looked at the role of gender, ethnicity, and post-traumatic stress symptoms in this relation. We collected data from 2612 students in New Haven, in the United States, over 2 years. The participants were from various ethnic backgrounds. We used surveys to measure their exposure to community violence and their post-traumatic stress symptoms in the first year, and in the second year, we measured their eating disorder symptoms. We found that adolescents who witnessed or were victims of community violence in the first year were more likely to have eating disorder symptoms in the second year. Post-traumatic stress symptoms seemed to explain most of this relation. Gender and ethnicity did not appear to change these findings in any meaningful way. Thus, It is likely important to assess and address post-traumatic stress symptoms when helping adolescents with eating disorders who have experienced community violence, regardless of their gender or ethnicity.

## Background

Eating disorders (ED), characterized by an over-evaluation of shape and weight, along with compensatory behaviors, leads to reduced quality of life and high medical risks [41, 53, 55, 63]. Despite current treatments, only 30–50% of patients benefit, and many relapse, emphasizing the importance of early identification and intervention [20, 51, 63, 64]. Thus, there is a need to identify individuals at risk of developing EDs, aiming to enhance interventions for this group.

The development of ED symptoms has been linked to a number of etiological factors, with genetics accounting for over 50% of the variance and environmental factors accounting for the remaining portion [22]. Among environmental factors, exposure to trauma has been identified as a substantial contributing factor in the development of ED symptoms and related adverse outcomes such as higher mortality and poorer quality of life [7, 25]. In particular, the negative effects of traumatic events such as exposure to sexual and physical violence, and emotional abuse on ED symptoms has been thoroughly investigated [4, 12, 38, 52, 56]. Importantly, despite the fact that interpersonal trauma represents a common type of traumatization among patients with EDs [31, 38], the effects of some forms of trauma on ED symptoms remains underexplored. This is especially the case as regards community violence exposure (CVE), which constitutes an extremely common type of violence exposure in adolescents [19, 57], and has been defined as the witnessing of, or victimization by, a violence-related act between individuals within one’s home, school, or neighborhood [11].

The most common psychiatric consequence of exposure to traumatic events is the development of posttraumatic stress (PTS) symptoms [62]. For individuals with PTS, a traumatic experience may entail long standing symptoms, such as disturbing flashbacks or dreams, avoidance, and distress in response to trauma-related ques [2]. PTS has, in turn, been associated with ED symptoms [6, 37], with approximately one in four of all ED patients also having a comorbid diagnosis of posttraumatic stress disorder (PTSD) [18, 49]. Further, it has been shown that the individuals with associated PTS show more severe ED psychopathology, comorbidity and quality of life in both adolescents [8] and adults [9], in contrast to the individuals with ED, but without PTS symptoms. The high prevalence of PTS in the ED population complicates interpretation of the association between traumatic events and ED symptoms as the development of trauma-related ED symptoms may be mediated through PTS. However, while the potential mediating effect of PTS in the association between interpersonal violence within a family or close relationship, such as childhood maltreatment (e.g., sexual and physical violence and emotional abuse) and disordered eating has been supported by previous research [26, 66], there has been a comparative lack of research exploring this effect with regard to the relation between CVE and ED symptoms. CVE differs from interpersonal violence in a family or close relationship in terms of the context where the violence occurs, i.e., it is not limited to personal relationships but can occur within a broader community or societal setting between unrelated individuals [67]. This addition to the field is important, as previous research has demonstrated that the type of trauma is associated with distinct effects and consequences [21, 30]. Consequently, potential differences in effect might necessitate diverse approaches in terms of e.g., assessment and treatment strategies.

It should also be noted that some demographic factors, such as gender and ethnic background might have an impact on both the development of ED symptoms, as well as the risk of CVE. Adolescents girls have an 8–10-fold higher risk of developing ED symptoms than boys [40], whereas CVE is more commonly reported by boys than girls [19, 29]. Moreover, previous research has shown that CVE is more common among ethnic minority adolescents residing in urban inner-city neighborhoods [13]. Research on differences in ED symptoms between ethnic minority and Caucasian groups has yielded somewhat disparate results, with some studies indicating there are differences [17, 50], while others have found that ethnic minority groups are affected by EDs as much as Caucasian groups [14, 32]. Nonetheless, it has been shown that disparities exist, with people in minority groups seeking help for EDs to a lesser extent and having a poorer response to treatment than Caucasians [1].

Against this background the objectives of the present study were: (1) to evaluate if CVE is related to ED symptoms in adolescents, (2) to investigate if the relation between CVE and ED symptoms is mediated by PTS, and (3) to explore whether the relation between CVE and ED symptoms is moderated by gender and ethnicity. We hypothesized that there would be a significant relation between CVE and ED symptoms and that this relation would be at least partially mediated by PTS symptoms. We further wanted to explore whether gender and ethnicity would moderate the relationship between CVE and ED symptoms, but due to the lack of previous research, no specific hypotheses were formulated in this regard.

## Methods

### Participants

The study participants comprised 3507 adolescents (1810 girls and 1697 boys) between the ages of 11 and 16 years old (*M* = 12.93, *SD* = 1.37) who were assessed in year one, and planned to be followed longitudinally over a period of one year. All participants were recruited from the public school system in New Haven, CT, in the United States. The data were collected within the framework of a large project, the Social and Health Assessment (SAHA). Further information on this project is available elsewhere [50]. Seventy-six percent of the original sample (*n* = 2664) completed the SAHA in year two. Respondents who reported “other” for ethnicity were excluded (*n* = 52) due to the heterogeneity with regard to ethnicity within this group. Specifically, it would not be appropriate to draw conclusions about this diverse group in the context of our research question on the moderating effect of ethnicity. After attrition and exclusion, 2612 participants remained and were included in the analyses. Please see Table 1 for sample characteristics. Similar to the present study, high attrition rates have been frequent in previous longitudinal studies with ethnic minority urban adolescents [42]. Those excluded due to missing data on ED symptoms after one year or ethnicity were somewhat older (*t* = 11.52, *p* < 0.001), more often boys (*χ*^*2*^ = 14.38, *p* < 0.001), and had a higher SES (*χ*^*2*^ = 10.85, *p* = 0.004). Effect size differences in age, gender, and SES for those excluded and those included were small to moderate. There was no statistical difference with regard to ethnicity (*χ*^*2*^ = 5.45, *p* = 0.065). For the 2612 included participants, only 0.8% of the values in the dataset were missing.

**Table 1:** Demographic characteristics of the 2612 adolescent students

Mean age (SD)	12.78 (1.29)
Age range	11–16 years
Gender *n* (%)	
Girls	1397 (53.5%)
Boys	1215 (46.5%)
Ethnicity *n* (%)	
Caucasian	358 (13.7%)
African American	1582 (60.6%)
Hispanic American	672 (25.7%)
Socio economic status *n* (%)	
No lunch discount	742 (28.4%)
Reduced lunch fee	200 (7.7%)
Free lunch	1670 (63.9%)

### Procedure

Data were collected longitudinally at two points in time (a baseline measure at year one and a follow up at year two) in all public schools in the city. Parents received information about the study when they registered their child for school in year one. In addition, information was posted to the parents two weeks prior to the time of the survey’s administration, where they also were free to decline their child’s participation in the study. The adolescents received information about the survey in their schools prior to its administration. They were read a detailed assent form outlining the participation conditions, and stating that their personal information would be kept confidential. Informed consent was obtained from all participants prior to the study. The adolescents could also decline to participate at the time the survey was administered. In all, parent and child refusals were less than 1%. The survey was completed during a regular class on a normal school day. For adolescents who were absent on the day of the survey, make-up data collections for absentee students were undertaken within one month of the initial survey. No compensation was offered for participation. The data collection procedure was repeated one year later. CVE, PTS symptoms and Socio-economic status (SES, ethnicity, gender and age) were assessed in year one. ED symptoms were assessed in year two. Ethical approval for the study was obtained from the institutional review board, Yale University School of Medicine.

### Instruments

#### Posttraumatic stress symptoms

The Child Post-Traumatic Stress—Reaction Index** (**CPTS-RI) [45, 46] is a 20-item self-rating questionnaire based on the Diagnostic and Statistical Manual of Mental Disorders, fourth edition (DSM-IV) that inquiries about reactions specific to current traumatic events. Answers were given using a 5-point Likert scale where the respondent indicated how often during the last 30 days, he/she had experienced PTS symptoms (e.g. “do you get scared or afraid because you think about bad things that have happened to you?”, “do thoughts or pictures of bad things that have happened to you come back to you, even when you don’t want them to?”): Never (scored 0), A little (1), Sometimes (2), Often (3), or Most of the time (4). Scores can range between 0 and 80, with those between 12 and 24 indicating mild PTSD, a score between 25 and 39 indicating moderate PTSD, a score between 40 and 59 indicating severe PTSD and scores of 60 or higher indicating very severe PTSD [46]. Previous research has shown the validity of using the CPTS-RI to detect PTSD [16, 46, 58]. In the current sample, the internal consistency of this measure was high with a Cronbach’s alpha value of 0.85.

#### Community violence exposure (CVE)

CVE was measured with two seven-item questionnaires designed to assess whether the respondents have seen someone being exposed to community violence (witnessing) or whether they themselves had been a victim of community violence (victimization) during the past year. Items were derived from the Screening Survey of Exposure to Community Violence Exposure developed by Martinez and Richters [48]. The following types of CVE were examined: being chased by gangs or individuals, threatened with serious physical harm, getting beaten up or mugged, attacked or stabbed with a knife, seriously wounded in an incident of violence, get shot or shot at with a gun, and threatened or harmed because of their race or ethnicity. Respondents answered using a five-point Likert scale to indicate how many times they had seen (witnessing) or had themselves been exposed to (victimization) community violence in the past year: none (scored 0), 1–2 times (1), 3–5 times (2), 6–9 times (3), 10 or more times (4). The scores were then summed to obtain a total score that could range between 0 and 28 for each scale. In line with previous research [34], in the present study, three groups were formed according to the reported types of exposure. (1) the *non-exposed group* (*n* = 565), i.e., those who did not report any witnessing or any victimization episodes, (2) the *witnessing group* (*n* = 1141), i.e., those who reported one or more episodes of witnessing, but no episodes of victimization, and 3) the *victimization group* (*n* = 906)*,* i.e., those who reported at least one episode of victimization. The validity of both scales has been previously supported by research showing that youth self-reports of exposure to violence overlap significantly with the location of violent criminal events as documented in official police reports [54].

#### Eating disorder symptoms

The Eating Problem Scale (EPS) is a six-item questionnaire that is a short version of the Eating Disorder Diagnostic Scale [59], which assesses ED symptoms during the past three months. The scale can be divided into two subscales: (1) *the occurrence of ED thoughts,* assessing symptoms related to an overvaluation of weight and shape during the past week, and (2) *the frequency of compensatory ED behaviors,* assessing the frequency of compensatory behaviors per week. The occurrence of ED thoughts subscale comprised the questions “I worried a lot about how to stop gaining weight”; “I felt fat even when others told me I am too thin”; “I ate large amounts of food even when I didn’t feel hungry” and “I felt very upset about my overeating or weight gain”. These questions were answered on a three-point scale: Not true (scored 0), Somewhat true (1), or Certainly true (2) providing a total score between 0 and 8. The compensatory ED behaviors subscale comprised the questions “About how many times per week have you made yourself vomit/used laxatives” and “About how many times per week have you fasted or engaged in excessive exercise”. These questions were answered on a five-point Likert scale: 0 times (scored 0), 1–2 times (1), 3–5 times (2), 6–10 times (3) and more than 10 times (4) yielding a total score ranging between 0 and 8. The internal consistency of this scale was acceptable in the present sample (Cronbach’s alpha was 0.74).

#### Socio-economic status

In the United States, government supported programs with reduced lunch fee or free lunch are administered by schools in order to assist families with lower income levels. To examine SES in the present sample, the proportion who received reduced or free lunch was therefore used as proxy for socio-economic disadvantage. SES was assessed in terms of receiving a lunch discount, where three groups were identified: (1) those who received no discount, (2) those who paid a reduced lunch fee, and (3) those who received free school lunches. At the time of data collection, free or reduced lunch were eligible for students whose family income was 185% or less of the federal poverty line.

### Statistics

As with most data in clinical research, missing data cannot be assumed to be missing completely at random. Therefore, missing data were handled through a process of multiple imputation, as recommended in previous studies [33, 43]. Five sets of imputations were generated, and the five datasets were subsequently pooled into one single dataset for the subsequent analyses.

To determine if a potential relation exists between our predictor and outcome variables we used the PROCESS macro developed by Hayes, v.4.1 for SPSS version 28.0.1.0 [23], a modified form of linear regression suitable for analyzing mediation and moderation. To test the hypothesis that CVE at year one and ED symptoms at year two were related (aim 1) as well as the hypothesis that the effect of CVE on ED symptoms behaviors was mediated by PTS symptoms measured at year one (aim 2), Hayes PROCESS macro (model 4) was utilized, as suggested appropriate with multicategorical independent variables and continuous mediating and dependent variables [24, 28]. Analyses were adjusted for gender, age, ethnicity, and SES. To test the hypothesis that gender and ethnicity moderated this effect (aim 3), Hayes PROCESS macro (model 5) was utilized. Analyses were adjusted for age and SES. All categorical variables were dummy coded. As the independent variable (CVE) is multicategorical and not continuous, the *no exposure* group was set as the reference category and contrasted to the *witnessing* and *victimization* groups. For the categorical variable ethnicity, *Caucasian* was set as the reference category and compared to *African American*, and *Hispanic American*. As Hayes macro is a form of linear regression, tests were performed to see if the data were suitable for these analyses. When we examined the normality of the sampling distribution it indicated that there were many zero values in the primary outcome measure which in some cases may be a problem. However, with Hayes model bootstrapping is used. Bootstrapping is a non-parametric resampling procedure and it is argued that the sampling distribution does not require normality as in the case of e.g., simple linear regression [44]. In addition, sensitivity analyses were also performed using negative binomial regression to further evaluate the accuracy of the findings in the mediation and moderation analyses in the PROCESS macro.

Power calculations indicated, given a power of 0.80, an alpha level at 0.05, with 9 predictors (CVE = 2, SES = 2, ethnicity = 2, PTS symptoms, gender, and age), that 791 participants would be required to identify a small effect size. Thus, the sample is sufficiently large for the analyses.

## Results

### Associations between community violence exposure and eating disorder symptoms

Table 2 shows the prevalence of ED symptoms and PTS symptoms by the degree of CVE. In model 1 the total effect of CVE on ED thoughts one year later (i.e., the effect without considering potential mediating or moderating effects) was significant when no exposure was compared to both witnessing and victimization (see the total effects column in Table 3). In model 2 the total effect of CVE on ED compensatory behaviors one year later was also significant when the no exposure group was compared to the victimization group, but not when compared to the witnessing group. The results are presented after controlling for gender, age, ethnicity, and SES.

**Table 2:** Descriptive statistics of eating disorder symptoms and posttraumatic stress symptoms (M(SD)) by the degree of CVE among 2612 adolescent students

	Degree of CVE		
	Non-exposed n = 565 (21.6%)	Witnessing n = 1141 (43.7%)	Victimization n = 906 (34.7%)
Eating disorder thoughts	1.65 (2.14)	1.88 (2.22)	1.94 (2.20)
Eating disorder compensatory behaviors	0.48 (1.10)	0.53 (1.06)	0.80 (1.46)
Posttraumatic stress symptoms	16.62 (11.18)	20.42 (11.59)	27.19 (14.33)

The values presented are not adjusted for the list of covariates

*CVE* Community violence exposure, *M* mean, *SD* standard deviation

**Table 3:** Mediation analyses of the relationship between CVE and eating disorder symptoms, with posttraumatic stress symptoms as mediator

Model	Total effects			Direct effects			Indirect effects		
	Coefficient (*b*-value)	*t*-value	*p*-value	Coefficient (*b*-value)	*t*-value	*p*-value	Coefficient (*SE*)	Percentile bootstrap 95% CI	
								Lower	Higher
Model 1: CVE on ED thoughts, no exposure vs witnessing	**.27**	**2.47**	**.014**	.17	1.52	.128	**.11 (.02)**	.07	.15
Model 1: CVE on ED thoughts, no exposure vs victimization	**.58**	**5.03**	** < .001**	.23	1.94	.052	**.35 (.05)**	.26	.44
Model 2: CVE on ED compensation, no exposure vs witnessing	.09	1.37	.17	.05	.80	.424	**.04 (.01)**	.02	.06
Model 2: CVE on ED compensation, no exposure vs victimization	**.36**	**5.39**	** < .001**	**.24**	**3.45**	** < .001**	**.12 (.03)**	.07	.17

Bold font indicate significant effects. For indirect effects, the effect is significant when the 95% confidence interval does not include zero

*CVE* Community violence exposure, *ED* Eating disorder, *CI* confidence interval

### The mediating effect of posttraumatic stress

**Fig. 1:**
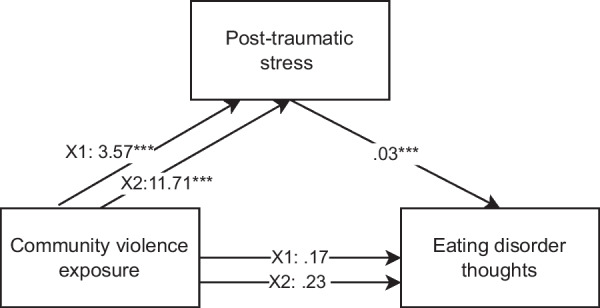
Mediation analysis examining the association between CVE and eating disorder thoughts, with posttraumatic stress symptoms as mediator. *Note* X1 represents the pairwise comparison between no exposure and witnessing, while X2 represents the pairwise comparison between no exposure and victimization

Mediation analyses were performed to test the potential mediating role of PTS symptoms in the association between CVE and ED symptoms. In model 1 (the effect of CVE on ED thoughts) results showed a significant indirect effect of the mediator PTS, indicating that mediation had occurred between both pairwise comparisons of no exposure and witnessing (X1) and no exposure and victimization (X2) (see also Fig. 1 and Table 3). As reported above, the total effect (i.e., the effect without the involvement of the mediator) of CVE on ED thoughts was significant. However, when the mediating variable PTS was included in the analysis, the direct effect of CVE on ED thoughts was no longer significant between no exposure and witnessing, or no exposure and victimization. Results are presented after controlling for gender, age, ethnicity, and SES.

**Fig. 2:**
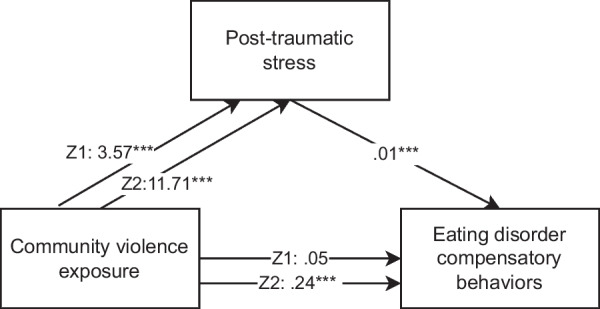
Mediation analysis examining the association between CVE and eating disorder compensatory behaviors, with posttraumatic stress symptoms as mediator. *Note* Z1 represents the pairwise comparison between no exposure and witnessing, while Z2 represents the pairwise comparison between no exposure and victimization

In model 2 (the effect of CVE on ED compensatory behaviors) there was a significant indirect effect of the mediator PTS symptoms, indicating that mediation had occurred between the pairwise comparisons of no exposure and witnessing (Z1) and no exposure and victimization (Z2) (see also Fig. 2 and Table 3). When the PTS mediating variable was included in the analysis, the direct effect of CVE on ED compensatory behaviors was not significant between no exposure and witnessing. However, there was still an association between no exposure and victimization when the PTS mediator was included. Results are presented after controlling for gender, age, ethnicity, and SES.

In the sensitivity analyses, the results from negative binomial regression analyses supported the findings from the mediation analyses. In the unadjusted (crude) model, CVE had a significant effect on ED thoughts. However, this effect disappeared in the adjusted model when PTS and other potential confounders were included in the analysis. Similarly, the results from negative binomial regression analyses also supported the findings from the mediation analyses for ED compensatory behaviors. Specifically, a significant unadjusted effect between CVE and ED compensatory behaviors was observed in the crude model, which remained significant in the adjusted model when PTS and potential confounders were included in the analysis.

### The moderating effect of gender and ethnicity

**Fig. 3:**
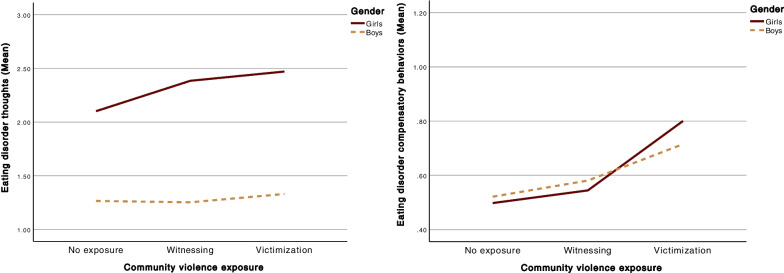
Visualization of the relation between CVE and eating disorder thoughts and compensatory behaviors respectively, moderated by gender

Gender significantly predicted ED thoughts (*b* = 0.84, *p* < 0.001), with girls (*M* = 2.38, *SD* = 2.40) displaying more ED thoughts than boys (*M* = 1.24, *SD* = 1.75). There was, however, no significant difference in ED compensatory behaviors (*b* = 0.02, *p* = 0.823) between girls (*M* = 0.61, *SD* = 1.18) and boys (*M* = 0.61, *SD* = 1.29). When investigating the moderating effect of gender in the association between CVE and ED thoughts, in the presence of PTS as a mediator and the potential confounders age, ethnicity, and SES, the effect was not significant: *R*^*2*^ = 0.001, 0.1%, *p* = 0.335 for ED thoughts, and *R*^*2*^ = 0.001, 0.1%, *p* = 0.515 for ED compensatory behaviors (see Fig. 3).

**Fig. 4:**
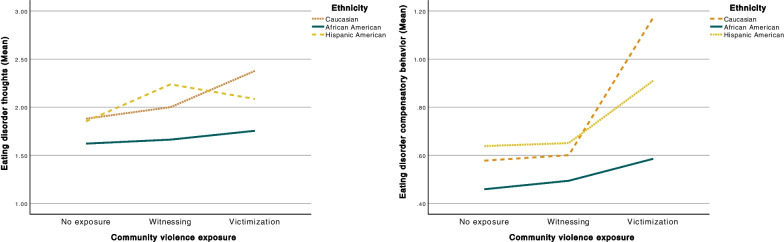
Visualization of the relation between CVE and eating disorder thoughts and compensatory behaviors respectively, moderated by ethnicity

Ethnicity did not predict ED thoughts, neither when comparing Caucasian and African American (*b* = 0.26, *p* = 0.258) or Caucasian and Hispanic American adolescents (*b* = 0.23, *p* = 0.401). The mean (*SD*) was 2.03 (2.40) for Caucasians, 1.71 (2.07) for African Americans, and 2.09 (2.34) for Hispanic Americans. Ethnicity also did not predict ED compensatory behaviors when comparing Caucasian and African American (*b* = 0.12, *p* = 0.374) or Caucasian and Hispanic American adolescents (*b* = 0.18, *p* = 0.135). The mean (*SD*) was 0.78 (1.75) for Caucasians, 0.52 (1.05) for African Americans, and 0.74 (1.34) for Hispanic Americans. When investigating the moderating effect of ethnicity in the association between CVE and ED symptoms, while including PTS as a mediator and age, gender, and SES as confounders, the effect was not significant for ED thoughts:* R*^2^ = 0.001, 0.1%, *p* = 0.385. However, it was significant for ED compensatory behaviors: *R*^2^ = 0.004, 0.4%, *p* = 0.03 (see Fig. 4). More specifically, there was one significant interaction effect: when victimization was contrasted with no exposure, Caucasians who were victimized had a significantly higher likelihood of engaging in ED compensatory behaviors than African Americans who were victimized.

## Discussion

The primary aim of this study was to evaluate the relation between CVE and ED symptoms one year later while investigating the potentially mediating effect of PTS symptoms and moderating effects of gender and ethnicity. Our main findings were that self-reported victimization at year one was related to both subsequent ED thoughts and compensatory behaviors one year later, whereas witnessing was only related to ED thoughts one year later. Additionally, the relationship between CVE and ED thoughts was fully mediated by PTS symptoms, whereas the occurrence of ED compensatory behaviors was only partially mediated by PTS. Thus, there seems to be a more direct association between victimization by community violence and ED compensatory behaviors, even when including PTS symptoms in the model. The above associations do not seem to be related to gender or ethnicity.

In line with our hypothesis, we found a significant relation between different types of CVE and ED symptoms, except for the relation between witnessing and ED compensatory behaviors, which was not significant. These results build on and extend previous findings that have linked ED symptoms to a number of other traumatic experiences, such as exposure to sexual and physical violence and emotional abuse as well as being exposed to accidents or other types pf trauma [4, 5, 9, 12, 36, 38, 52, 56]. The exact mechanisms underlying the association between trauma and EDs are however uncertain, although various factors, such as emotion dysregulation and dissociation, may be involved [65].

Consistent with previous research on other types of trauma, such as child maltreatment [26, 66], our study suggests that PTS symptoms may have an important mediating role in the association between CVE and ED symptoms, which was found for both ED thoughts and ED compensatory behaviors. These findings further support the idea that ED symptoms may serve to regulate trauma-related negative effects [26, 27] among individuals with ED symptoms who have been exposed to potentially traumatic events such as CVE. Findings indicate that it is likely beneficial to screen trauma in individuals with EDs. Furthermore, for those exposed to community violence, it is likely to be important to direct treatment efforts not only towards ED symptoms, but also towards symptoms of trauma, as they may persist and impact treatment outcomes, potentially leading to a relapse in ED symptoms [26, 66]. Interestingly, the relation between CVE and ED thoughts was fully explained by PTS symptoms, while ED compensatory behaviors were only partially explained by them. It can be speculated that ED compensatory behaviors may represent a more general, non-specific maladaptive coping mechanism related to coping with trauma [37], above and beyond PTS symptoms.

Previous studies have consistently shown that girls tend to have more severe ED thoughts than boys [15, 47], which is consistent with our findings. However, our study did not find any evidence that gender moderates the relationship between CVE and ED symptoms, when mediated by PTS. Neither did we find any substantial support for the idea that ethnicity would moderate the relation between CVE and ED symptoms, with the exception of Caucasian adolescents who reported more severe ED compensatory behaviors than African Americans in the victimized group, in contrast to the no exposure group. It should, however, be noted, that as several comparative moderation analyses were performed, this finding might simply be due to chance and therefore is in need of replication. Overall, the main clinical implication of the moderation analysis is that neither gender nor ethnicity is likely to be important to consider when planning the care of individuals with ED symptoms that have been exposed to community violence.

The study has several strengths. In particular, data came from a large cohort of inner-city, mostly ethnic-minority youths that were obtained within the framework of a longitudinal survey. However, it should be noted that the study also has a number of limitations. First, even though the study had a longitudinal design with two measurement points, ED symptoms were not assessed in year one, making it inappropriate to draw any causal conclusions on whether changes in ED symptoms were due to CVE. Additional measurement points assessing both PTS symptoms and ED symptoms beyond the follow-up after one year would have further strengthened the study. Second, all the measures were based on self-ratings. Even though self-rated health data reported by adolescents are generally valid [39], data from other sources, including diagnostic interviews, would have strengthened the overall study. With self-ratings, we can only draw conclusions regarding self-observed ED symptoms as well as PTS symptoms, while we cannot draw any firm conclusion on whether our findings also apply to e.g., clinically rated ED or PTSD diagnoses. Importantly, the scale measuring PTS symptoms (CPTS-RI) does not provide the information necessary for a formal PTSD diagnosis. The CPTS-RI is also based on DSM-IV offering details closely aligned with symptoms related to PTS in DSM-IV. Despite disparities between DSM-IV and DSM-5, such as an increased focus on behavioral symptoms and alterations in how the symptom clusters [3], both editions encompass core criteria for PTSD. These criteria involve for example avoidance, negative alterations in mood and cognitions, and changes in reactivity—symptoms assessed using the scale in this study. Third, there are several characteristics that might be relevant to the risk of developing ED symptoms and their relation to CVE, such as heritability [61] or emotion regulation deficits [60], that we did not have information on and therefore could not examine. Further, the study did not have any information regarding sexual orientation or gender identity. This would have made an important contribution to the field, as sexual and gender minority individuals represent up to one fourth of all patients with EDs [10], and report both higher levels of trauma and more severe ED symptoms [35]. Also, even though lunch discount is a quite solid proxy for financial difficulties, SES is a multifaceted concept that includes factors beyond income, such as education and occupation. Fourth, PTS symptoms were not assessed specifically in relation to CVE, and may therefore correspond to other traumatic events. Finally, these data came from one small area in the United States and thus might not be representative of most boys and girls. The age range of the study participants (11 and 16 years of age) also limits generalizability of the findings.

## Conclusions

In conclusion, we found that both witnessing of and victimization by community violence were related to ED symptoms, with most or all of these associations being explained by PTS symptoms. Neither gender nor ethnicity seem to have any substantial effect on this relation. These findings indicate that detecting and addressing PTS symptoms might be beneficial when treating adolescents with ED symptoms who have been exposed to community violence. This has, however, not been investigated in this study and further research should evaluate the longitudinal effects of interventions that provide support for either ED symptoms, PTS symptoms, or both.

## Data Availability

The datasets used in this article are not readily available because the data cannot be shared publicly due to the initial decision of the local ethical committee, as well as the restrictions included in the informed consent statement (where it was stated that the data would only be used by the research group and would not be transferred elsewhere).
